# Two Rare Diseases, One Patient: A Case Report of Mucormycosis and Granulomatous Polyangiitis

**DOI:** 10.1155/2023/7934700

**Published:** 2023-05-10

**Authors:** Syeda Aasia Batool, Usha Kumari, Salim Surani

**Affiliations:** ^1^Holy Family Hospital, Rawalpindi, Pakistan; ^2^Dow University of Health Sciences, Karachi, Pakistan; ^3^Medicine & Pharmacology, Texas A&M University, College Station, Texas, USA

## Abstract

Mucormycosis is a rare but fatal disease caused by a filamentous fungus involving the nose, paranasal sinuses, and brain. These organisms usually cause severe infections in immunocompromised individuals. Granulomatous polyangiitis (GPA), also known as Wegner's granulomatosis, is a rare, aseptic necrotizing granulomatous vasculitis involving small and medium-sized vessels commonly affecting the nose, ears, lungs, and kidneys. The simultaneous occurrence of mucormycosis and GPA, two rare diseases, in the same patient is exceedingly rare. In this case study, we describe a 40-year-old woman who presented with manifestations of both GPA and mucormycosis. She was started with steroids and antifungal agents and achieved significant improvement.

## 1. Introduction

Mucormycosis is a necrotizing, destructive disease. Immunocompromised individuals are frequently affected by it, including those with acquired immunodeficiency syndrome (AIDS), hemochromatosis, severe burns, prolonged steroid therapy, cancer, and diabetic ketoacidosis. However, some studies show the presence of mucormycosis without any predisposing factor [1]. Granulomatosis with polyangiitis (GPA) is a rare multisystem necrotizing vasculitis, mostly involving the upper respiratory tract, lungs, and kidneys. It is a type of antineutrophil-cytoplasmic-antibody (ANCA)-associated vasculitis (AAV). The global incidence of GPA is approximately 10–20 cases per one million annually [2] with no gender predisposition but mainly occurs in the older population [2, 3]. Diagnosis requires thorough and focused history, a clinical exam, an autoimmune profile, and radiological and histopathological analyses [2]. The treatment involves immunosuppressive agents in two phases, i.e., the induction and maintenance phases [2]. The average life expectancy of GPA without any treatment is five months [2].

Both GPA and mucormycosis are rapidly progressive and potentially fatal diseases, so early diagnosis and prompt appropriate treatment are required for a better prognosis [1, 2]. In this case study, a patient with a GPA diagnosis was found to have mucormycosis. Because these are two distinct rare disorders, and their co-occurrence in a single patient is even more unusual, further research is required to understand the underlying mechanisms and appropriately manage these rare but complicated diseases.

## 2. Case Presentation

**Figure 1:**
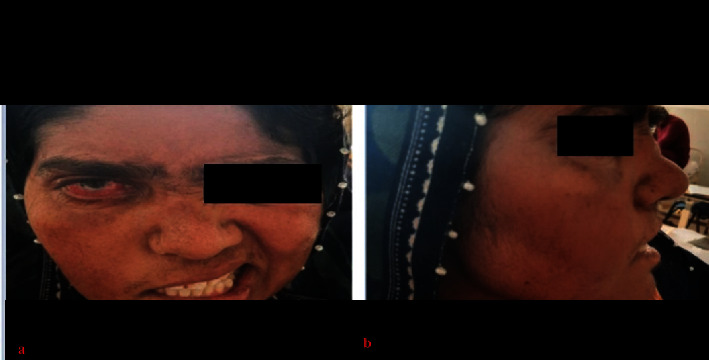
Facial anomality in patient (a) facial swelling (b) nasal deformity.

A 40-year-old Asian female with no known comorbidity presented to the outpatient department with complaints of progressive bilateral loss of hearing and a runny nose for four weeks. A high-grade fever was documented up to 103°F with a productive cough and dull frontal headache for one week. Previously, she had been treated for sinusitis and otitis media in a local hospital with various antibiotic regimens and antihistamines, with no notable improvement. Therefore, the patient was referred to us at the tertiary care hospital. On physical examination, she had right-sided facial swelling, right eye proptosis with conjunctival redness, saddle-shaped nose deformity, bilateral polypoid growth, right-sided lower motor type of facial nerve palsy (Figures 1(a) and 1(b)), and sensorineural hearing loss on the same side. On auscultation of the left lung, crackles with decreased breath sounds were noted. The rest of the systemic examination was unremarkable. The patient was admitted to the medicine ward.

**Figure 2:**
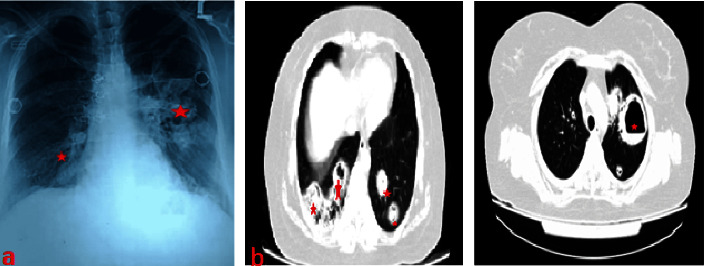
(a) X-ray chest (b) high-resolution computerized tomography (HRCT).

**Figure 3:**
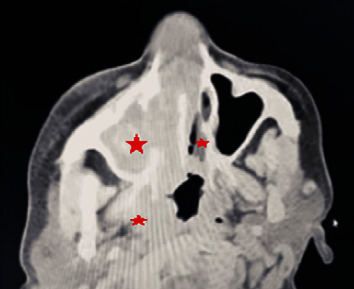
Computerized tomography (CT)-scan paranasal sinuses with sinuses with the area shown in the red star reveal nodular mucosal thickening in bilateral nasal cavities with widening and ^*∗*^showing destruction of right osteomeatal complex and pansinusitis.

**Figure 4:**
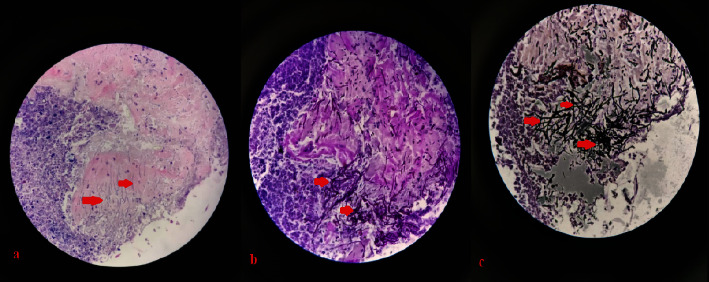
(a) H&E stain, (b) periodic acid-Schiff (PAS) stain, (c) grocott methamine silver stain showing long fibers of broad non-septate hyphae branching at 90 degrees (red arrows).

The complete laboratory workup is shown in Table 1. Anteroposterior (AP) chest X-ray revealed bilateral multiple nodular opacities and thick-walled cavities (Figure 2(a)). High-resolution computerized tomography (HRCT) showed bilateral multiple large cavitating soft tissue nodules with some air-fluid levels (Figure 2(b)), and computerized tomography (CT)-scan paranasal sinuses revealed nodular mucosal thickenings in bilateral nasal cavities with widening and destruction of right osteomeatal complex and pansinusitis (Figure 3). A nasal mucosa biopsy revealed a necrotic slough with wide-angle branching fungal hyphae suggestive of mucormycosis (Figure 4).

**Figure 5:**
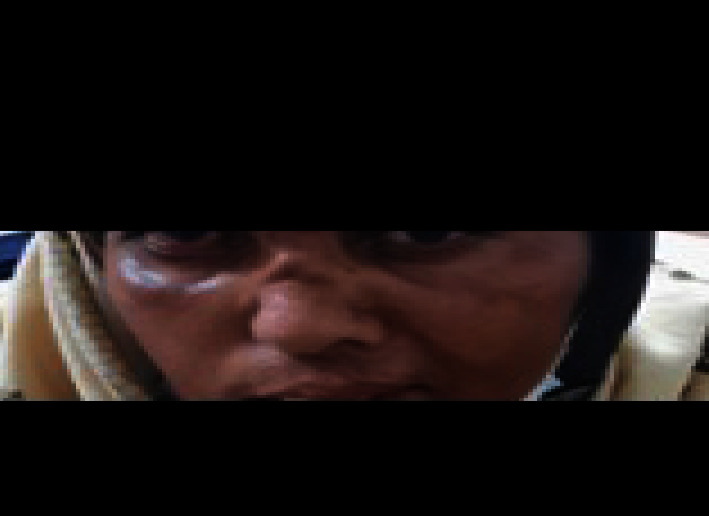
The patient's facial image shows marked improvement on a follow-up visit.

A diagnosis of GPA with coexisting mucormycosis was made. Treatment started with intravenous (IV) methylprednisolone 1000 mg for three days (pulse therapy), followed by an oral steroid of 50 mg/day, and slow infusion of liposomal amphotericin B (LAm-B) 3 mg/kg/day in two divided doses for two weeks. There were marked improvements in proptosis and hearing. Thus, she was started on injectable cyclophosphamide (CYC) 1 gm undercover of mesna. The patient showed progressive improvement and was discharged after 20 days. The patient received a monthly IV CYC 1 Gram daily under cover of mesna for three months, LAm-B 3 mg/kg/day in 2 divided doses for a month, and oral steroids, which were tapered off gradually.  Figure 5 shows improved facial symptoms on a follow-up visit. Laboratory workup on follow-up showed improved inflammatory markers and renal function tests.

## 3. Discussion

Mucormycosis can mimic relapse or progression of GPA. It is crucial to timely diagnose and treat mucormycosis infection in order to prevent a disseminated disease with a poor prognosis [4]. There are very few reported cases of mucormycosis in patients with GPA [5, 6]. It may suggest an underdiagnosis of mucormycosis as the symptoms closely mimic the GPA relapse. Furthermore, many such cases may have been treated as GPA relapse with a poor prognosis [5]. We present a rare case of a patient with concomitant mucormycosis and GPA.

Mucormycosis is a rare, necrotizing, rapidly progressive disease due to its angioinvasive pathogenesis [1, 7]. Organism invades blood vessels via endothelial damage leading to a blood clot and occlusion of vessels which in turn causes ischemia and necrosis [1, 7]. It spreads via fungal spores inhalation [1] and primarily causes infection in only immunocompromised individuals. However, the absence of predisposing risk factors does not exclude mucormycosis. Research showed that about 9% of rhinocerebral mucormycosis diagnoses in patients without any predisposing factors [1]. Despite optimal treatment, this infection has a 50% mortality rate [7].

Mucormycosis is generally diagnosed through tissue biopsy, which reveals broad, nonseptate hyphae with right-angle branching [1, 7]. Alternatively, a noninvasive and rapid method, quantitative PCR (qPCR) detection of mucorales DNA in serum, might be performed as a first test if there is clinical suspicion of mucormycosis [8].

Surgical debridement of necrotic tissue and amphotericin B (AMB) is the treatment of choice for mucormycosis [1, 9]. In case of AMB toxicity or preexisting kidney disease, isavuconazole and posaconazole can be used [4, 9]. Blood vessel occlusion and thrombosis are potential complications of mucormycosis. Hyperbaric oxygen therapy (HBO) could resolve granulation formulation and bone healing via increased vascularity and angiogenesis [9].

GPA is a rare aseptic necrotizing granulomatous vasculitis of small and medium-sized blood vessels belonging to a group of antineutrophil cytoplasmic antibody (ANCA)-associated vasculitis (AAV) [2, 3]. Cytoplasmic-ANCA (c-ANCA) is the most specific marker for GPA and is seen in 80–90% of GPA cases [2]. Combination therapies using glucocorticoids and CYC [2, 3], or rituximab and CYC [3] have shown to be successful. Moreover, plasmapheresis is indicated in patients with severe disease that is unresponsive to intravenous glucocorticoids [2]. The mortality rate within one year is significantly higher in patients with systemic vasculitis due to adverse events associated with therapy (59%) compared to active vasculitis itself [6]).

Patient with mucormycosis and GPA can present with varying severity. In our case, patient was successfully managed with debridement and conservative management. However, there have been cases of GPA with mucormycosis requiring otorhinolaryngological surgical procedures [4]. Because both diseases have overlapping symptoms and catastrophic outcomes in untreated individuals, a comprehensive workup is required to distinguish both conditions and treat them effectively. Starting immunosuppressive treatment for GPA without simultaneously treating the fungal infection can result in disseminated mucormycosis, which has a poor prognosis. As a result, a thorough evaluation is critical for a successful outcome [4].

## Figures and Tables

**Table 1:** Laboratory data.

Investigation	Value
*Complete blood count (CBC)*	
Hemoglobin	13.0 grm/dl
White blood cells	12.9 (10^9^/L)
Platelets	293,000

*Inflammatory markers*	
C-reactive protein	48.6 mg/l
Erythrocyte sedimentation rate (ESR)	75 ml/hr

*Renal function tests*	
Creatine	1.8 mg/dl
Urea	46 mg/dl
24-hour urinary protein	0.7 gm per 24 hours
Egfr	33 ml/min/73 m^2^

*Autoimmune work up*	
c-ANCA	Positive
p-ANCA	Negative
C3	2.1
C4	0.76
Antinuclear antibody (ANA)	Negative

*Miscellaneous*	
Glycated hemoglobin (HbA1c)	5.3%
Blood culture	No growth
COVID-19 polymerase chain reaction (PCR)	Negative
Sputum for AFB gene expert	MTB not detected
Hepatitis B and C by ELISA	Negative
HIV antigen/antibody	Negative

e-GFR; estimated glomerular filtration rate; c-ANCA; cytoplasmic-antineutrophilic cytoplasmic antibody, p-ANCA; perinuclear-antineutrophilic cytoplasmic antibody, MTB; mycobacterial tuberculosis, ELISA; enzyme linked immunosorbent assay, HIV; Human immunodeficiency virus.

## Data Availability

Available on request.
